# Supplementary material for: Resonating holes vs molecular spin-orbit coupled states in group-5 lacunar spinels
Source: Nat Commun. 2023 Aug 26;14:5218. doi: 10.1038/s41467-023-40811-y (PMC10460446; doi:10.1038/s41467-023-40811-y)
Supplement: Supplementary file 1 — Supplementary Information [file 41467_2023_40811_MOESM1_ESM.pdf]

Supplementary Information

Resonating holes vs molecular spin-orbit coupled states in group-5 lacunar spinels

Petersen *et al.*

CONTENTS

I. Supplementary Methods 1
A. Embedded cluster protocol 1
B. Computational Methods 1

II. Supplementary Table: Low-energy excitation energies 3

III. Supplementary Table: Leading ground state configurations 4

IV. Supplementary Table: Calculated dynamical $I_{\rm D}$ and nondynamical $I_{\rm ND}$ indices 5

V. Supplementary Figure: Calculated Susceptibility Curves 6

Supplementary References 7

I. SUPPLEMENTARY METHODS

A. Embedded cluster protocol

The quantum mechanical cluster model $[M_4X_{28}A_6]^{25-}$ (with M = V, Nb, Ta X = S, Se and A = Al, Ga) was embedded in a field of point charges (PCs), which was created using the EWALD program [1, 2] and experimentally determined geometries for the high-temperature cubic phase [3–6]. To initialize the EWALD optimization, the initial charges given in Supplementary Tab. 1 were employed. Those were determined under two constraints:

(1) the difference between the modified charges of the reciprocal unit cell and the formal charges necessary for extracting the quantum cluster is zero and

(2) the difference between these initial charge values and to atomic charges of the quantum cluster fitted to the molecular electrostatic potential (CHELPG, charges from electrostatic potentials using a grid-based method [7–10]) is minimal.

Between optimized PCs and quantum cluster, a total of 60 atoms (48 M, 12 X_i) were equipped with pseudopotentials for V from Dolg *et al.* [11], for Nb and Ta from from Andrae *et al.* [12], and for S/Se from Bergner *et al.* [13]. For $GaNb_4Se_8$ and $GaTa_4Se_8$ in particular, also smaller $[M_4Se_{16}]^{19-}$ cluster models were used to make computationally demanding calculations (NEVPT2 in particular) feasible. Here, the outermost Ga (6 atoms) and Se_o (12 atoms) were assigned cECPs from Leininger *et al.* [14] and Bergner *et al.* [13] with the appropriate charges given in Supplementary Table 1, respectively.

Supplementary Table 1. Initial point charge values for the employed quantum cluster models. X_i and X_o refer to X ligand atoms inside and outside of the $V_4/Nb_4/Ta_4$ unit. Additionally, the reference used for the HT-phase crystal structure is given.

Compound	Quantum cluster	Ga/Al	V/Nb/Ta	$S_\mathrm{i}/Se_\mathrm{i}$	$S_\mathrm{o}/Se_\mathrm{o}$	Ref. HT-cryst.
GaV_4S_8	$[V_4S_{28}Ga_6]^{25-}$	1.48	2.07	−0.82	−1.62	[3]
GaV_4Se_8	$[V_4Se_{28}Ga_6]^{25-}$	1.48	2.07	−0.82	−1.62	[3]
AlV_4S_8	$[V_4S_{28}Al_6]^{25-}$	1.48	2.07	−0.82	−1.62	[4]
$GaNb_4S_8$	$[Nb_4S_{28}Ga_6]^{25-}$	1.40	2.05	−0.80	−1.60	[5]
$GaNb_4Se_8$	$[Nb_4Se_{28}Ga_6]^{25-}$	1.40	2.05	−0.80	−1.60	[6]
	$[Nb_4Se_{16}]^{19-}$	3.00	2.00	−0.75	−2.00	[6]
$GaTa_4Se_8$	$[Ta_4Se_{28}Ga_6]^{25-}$	1.40	2.05	−0.80	−1.60	[6]
	$[Ta_4Se_{16}]^{19-}$	3.00	2.00	−0.75	−2.00	[6]

B. Computational Methods

Our study is based on the complete active space self-consistent field (CASSCF) approach [15], in which the overall molecular orbital space is divided into three parts: an inactive, an active and a virtual space. While inactive (doubly-occupied) and virtual (empty) space are optimized in a mean-field Hartree-Fock (HF) manner, a predefined set of N electrons in M orbitals are chosen as active, denoted as CAS(N,M). In this active space, stationary energy levels are optimized not only with respect to variations in the molecular orbital (MO) but also in configuration interaction (CI) coefficients. This essentially corresponds to an exact diagonalization within this subspace. Additionally, to account for dynamical correlation effects (i.e. electron-electron repulsion beyond the mean-field approximation) the N-electron valence perturbation 2nd order perturbation theory (NEVPT2) [16] was applied on top of the CASSCF wavefunction to provide a correction for the multiplet splittings.

The CASSCF approach [15] is fully *ab initio* – therefore, it is crucial to choose the basis sets of the atoms in the quantum cluster appropriately. While large basis sets are computationally too demanding, small basis sets do not yield a satisfactorily accuracy, especially beyond the mean-field approximation. A sufficient accuracy-speed trade-off was found for triple-ζ valence polarized (TZVP) basis sets on central and double-ζ basis sets on outer

atoms, respectively. For each atomic species, the employed basis sets are given in the following Supplementary Table 2. Since a strong effect of spin-orbit coupling (SOC) on the electronic states was found in both $GaNb_4Se_8$ and $GaTa_4Se_8$ [17–19], we additionally enable the Douglas-Kroll-Hess (DKH) approximation [20, 21] and used appropriately decontracted basis set variants.

Supplementary Table 2. Basis set per atom of each quantum cluster. X_i (with X = S, Se) refer to atoms directly bonding to the $[M_4]^{13+}$ cluster (16 atoms), while X_o atoms are the outermost atoms bonding to Ga/Al (12 atoms) (c.f. Fig. 1 in the manuscript). "$\sum$" denotes the overall number of contracted basis functions. The reference citation is also given.

Quantum cluster	Atom	Basis set	Ref.	Quantum cluster	Atom	Basis set	Ref.
$[V_4S_{28}Ga_6]^{25-}$	V	cc-pVTZ-DK	[22]	$[V_4Se_{28}Ga_6]^{25-}$	V	cc-pVTZ-DK	[22]
	S_i	cc-pVTZ-DK	[23]		Se_i	cc-pVTZ-DK	[23]
	Ga	cc-pVDZ-DK	[23]		Ga	cc-pVDZ-DK	[23]
	S_o	cc-pVDZ-DK	[23]		Se_o	cc-pVDZ-DK	[23]
	$\sum$	1194			$\sum$	1446	
$[V_4S_{28}Al_6]^{25-}$	V	cc-pVTZ-DK	[22]	$[Nb_4S_{28}Ga_6]^{25-}$	Nb	SARC-DKH-TZVPP	[24]
	S_i	cc-pVTZ-DK	[23]		S_i	DKH-DEF2-TZVPP	[25]
	Al	cc-pVDZ-DK	[23]		Ga	DKH-DEF2-SVP	[25]
	S_o	cc-pVDZ-DK	[23]		S_o	DKH-DEF2-SVP	[25]
	$\sum$	1140			$\sum$	1556	
$[Nb_4Se_{28}Ga_6]^{25-}$	Nb	SARC-DKH-TZVPP	[24]	$[Ta_4Se_{28}Ga_6]^{25-}$	Ta	SARC-DKH-TZVPP	[26]
	Se_i	DKH-DEF2-TZVPP	[25]		Se_i	DKH-DEF2-TZVPP	[25]
	Ga	DKH-DEF2-SVP	[25]		Ga	DKH-DEF2-SVP	[25]
	Se_o	DKH-DEF2-SVP	[25]		Se_o	DKH-DEF2-SVP	[25]
	$\sum$	2124			$\sum$	2212	
$[Nb_4Se_{16}]^{19-}$	Nb	SARC-DKH-TZVPP	[24]	$[Ta_4Se_{16}]^{19-}$	Ta	SARC-DKH-TZVPP	[26]
	Se_i	DKH-DEF2-TZVPP	[25]		Se_i	DKH-DEF2-TZVPP	[25]
	Se_o	DKH-DEF2-TZVPP	[25]		Se_o	DKH-DEF2-TZVPP	[25]
	$\sum$	1368			$\sum$	1456	

In Supplementary Fig. 1, the active space orbitals of the employed CAS(7e,12o) are depicted in natural (1(a)) and localized orbital (1(b)) representations. For the former, the corresponding irreps according to T_d point group symmetry are also given.

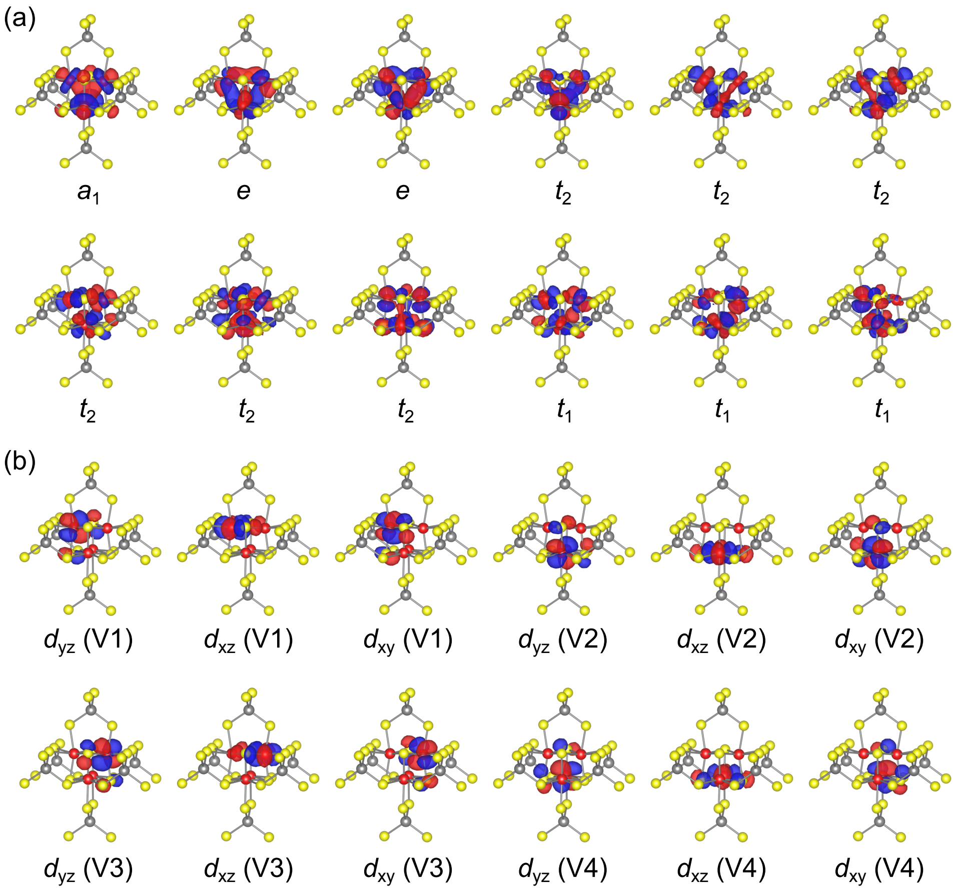

Supplementary Figure 1. Active space orbitals used in CAS(7e,12o). (a) Natural orbital representation. (b) Localized orbital representation.

II. SUPPLEMENTARY TABLE: LOW-ENERGY EXCITATION ENERGIES

Supplementary Table 3. Excitation energies (meV) of GaV_4S_8 using a $[V_4S_{28}Ga_6]^{25-}$ cluster model (CAS(7e,12o)). Three sextets, six quartets and seven doublets were included in the state-average procedure. Notation according to T_d point group symmetry. For each leading configuration its respective weight in the overall wavefunction is given.

State	Leading config.	E_{CASSCF}	E_{NEVPT2}	$E^{\text{SOC}}_{\text{NEVPT2}}$
$^2T_2$	$a_1^2\, e^4\, t_2^1\, t_1^0\, t_2^0$ (21%)	134	0	0 ($J = 3/2$), 12 ($J = 1/2$)
$^4T_1$	$a_1^2\, e^3\, t_2^2\, t_1^0\, t_2^0$ (25%)	0	41	38, 42, 43, 48, 48, 50
$^4T_1$	$a_1^2\, e^3\, t_2^2\, t_1^0\, t_2^0$ (24%)	44	82	80, 84, 85, 88, 89, 89
^6E	$a_1^1\, e^3\, t_2^3\, t_1^0\, t_2^0$ (47%)	31	125	129 (3 KD's), 130 (3 KD's)
$^6A_2$	$a_1^2\, e^2\, t_2^3\, t_1^0\, t_2^0$ (36%)	93	143	147 (3 KD's)
$^2A_1$	$a_1^2\, e^3\, t_2^2\, t_1^0\, t_2^0$ (25%)	90	164	168
^2E	$a_1^2\, e^3\, t_2^2\, t_1^0\, t_2^0$ (23%)	98	173	177 (2 KD's)
$^2A_1$	$a_1^2\, e^3\, t_2^2\, t_1^0\, t_2^0$ (25%)	140	207	212

Supplementary Table 4. Excitation energies (meV) of $GaNb_4Se_8$ using a large $[Nb_4Se_{28}Ga_6]^{25-}$ and a small $[Nb_4Se_{16}]^{19-}$ cluster model (CAS(7e,12o)). The later was used to make highly-demanding NEVPT2 calculations feasible. Three sextets, six quartets and seven doublets were included in the state-average procedure. Notation according to T_d point group symmetry. For each leading configuration its respective weight in the overall wavefunction is given.

		$[Nb_4Se_{28}Ga_6]^{25-}$		$[Nb_4Se_{16}]^{19-}$		
State	Leading config.	E_CASSCF	$E^\mathrm{SOC}_\mathrm{CASSCF}$	E_CASSCF	E_NEVPT2	$E^\mathrm{SOC}_\mathrm{NEVPT2}$
$^2T_2$	$a_1^2\,e^4\,t_2^1\,t_1^0\,t_2^0$ (64%)	0	0 ($J=3/2$)	0	0	0 ($J=3/2$)
			97 ($J=1/2$)			98 ($J=1/2$)
$^4T_2$	$a_1^2\,e^3\,t_2^2\,t_1^0\,t_2^0$ (70%)	478	475−522	473	496	492−537
$^4T_1$	$a_1^2\,e^3\,t_2^2\,t_1^0\,t_2^0$ (69%)	651	653−702	648	648	649−698
$^2T_1$	$a_1^2\,e^3\,t_2^2\,t_1^0\,t_2^0$ (67%)	832	835−881	826	779	783−838
$^6A_1$	$a_1^2\,e^2\,t_2^3\,t_1^0\,t_2^0$ (76%)	862	895	851	816	848
$^2A_2$	$a_1^2\,e^3\,t_2^2\,t_1^0\,t_2^0$ (70%)	869	905	869	923	963
^2E	$a_1^2\,e^3\,t_2^2\,t_1^0\,t_2^0$ (67%)	882	920	879	904	943
$^2A_1$	$a_1^2\,e^3\,t_2^2\,t_1^0\,t_2^0$ (62%)	884	924	881	883	923
$^2T_2$	$a_1^2\,e^3\,t_2^2\,t_1^0\,t_2^0$ (67%)	985	1008−1040	981	873	894−921
^2E	$a_1^2\,e^3\,t_2^2\,t_1^0\,t_2^0$ (63%)	1059	1100	1053	1059	1107

Supplementary Table 5. Excitation energies (meV) of $GaTa_4Se_8$ using a large $[Ta_4Se_{28}Ga_6]^{25-}$ and a small $[Ta_4Se_{16}]^{19-}$ cluster model (CAS(7e,12o)). The later was used to make highly-demanding NEVPT2 calculations feasible. Three sextets, six quartets and seven doublets were included in the state-average procedure. Notation according to T_d point group symmetry. For each leading configuration its respective weight in the overall wavefunction is given.

		$[Ta_4Se_{28}Ga_6]^{25-}$		$[Ta_4Se_{16}]^{19-}$		
State	Leading config.	E_CASSCF	$E^\mathrm{SOC}_\mathrm{CASSCF}$	E_CASSCF	E_NEVPT2	$E^\mathrm{SOC}_\mathrm{NEVPT2}$
$^2T_2$	$a_1^2\,e^4\,t_2^1\,t_1^0\,t_2^0$ (71%)	0	0 ($J=3/2$)	0	0	0 ($J=3/2$)
			345 ($J=1/2$)			347 ($J=1/2$)
$^4T_2$	$a_1^2\,e^3\,t_2^2\,t_1^0\,t_2^0$ (77%)	639	598−717	629	632	588−683
$^4T_1$	$a_1^2\,e^3\,t_2^2\,t_1^0\,t_2^0$ (77%)	864	880−1042	859	811	834−993
$^2T_1$	$a_1^2\,e^3\,t_2^2\,t_1^0\,t_2^0$ (75%)	994	1046, 1192	985	912	979, 1104
$^2A_2$	$a_1^2\,e^3\,t_2^2\,t_1^0\,t_2^0$ (77%)	1114	1261	1111	1128	1289
^2E	$a_1^2\,e^3\,t_2^2\,t_1^0\,t_2^0$ (75%)	1128	1319	1123	1104	1274
$^2A_1$	$a_1^2\,e^3\,t_2^2\,t_1^0\,t_2^0$ (69%)	1139	1305	1132	1068	1234
$^2T_2$	$a_1^2\,e^3\,t_2^2\,t_1^0\,t_2^0$ (76%)	1159	1234, 1370	1153	1011	1128, 1208
$^6A_1$	$a_1^2\,e^2\,t_2^3\,t_1^0\,t_2^0$ (83%)	1177	1293	1156	1093	1209
^2E	$a_1^2\,e^3\,t_2^2\,t_1^0\,t_2^0$ (71%)	1296	1587	1289	1251	1541

III. SUPPLEMENTARY TABLE: LEADING GROUND STATE CONFIGURATIONS

In the following Supplementary Table 6, the composition of the ground state $^2T_2$ term for each of the investigated lacunar spinel compounds in terms of electronic configurations with associated weights are given. These electronic configurations are given based on natural (molecular-like picture) and localized (atomic-like) orbitals. From both representations, it becomes clear that the $3d$-vanadate and the $4d/5d$-lacunar spinels form two groups that differ strongly in the amount of mixing of the leading $a_1^2e^4t_2^1t_1^0t_2^0$ (in molecular orbitals) or $t_{2\mathrm{g}}^1t_{2\mathrm{g}}^2t_{2\mathrm{g}}^2t_{2\mathrm{g}}^2$ (in localized orbitals) configurations.

Supplementary Table 6. Leading electronic configurations and associated weights of the ground state term in the investigated group-5 compounds. Both natural (molecular-like) and localized (atomic-like) orbital basis are given.

Compound	Molecular	Localized	Compound	Molecular	Localized
GaV_4S_8	$a_1^2e^4t_2^1t_1^0t_2^0$ (21%)<br>$a_1^1e^3t_2^3t_1^0t_2^0$ (6%)<br>$a_1^2e^2t_2^1t_1^1t_2^1$ (3%)<br>...	$t_{2\mathrm{g}}^1t_{2\mathrm{g}}^2t_{2\mathrm{g}}^2t_{2\mathrm{g}}^2$ (88%)<br>$t_{2\mathrm{g}}^1t_{2\mathrm{g}}^2t_{2\mathrm{g}}^1t_{2\mathrm{g}}^3$ (11%)<br>...	GaV_4Se_8	$a_1^2e^4t_2^1t_1^0t_2^0$ (18%)<br>$a_1^1e^3t_2^3t_1^0t_2^0$ (6%)<br>$a_1^2e^2t_2^1t_1^1t_2^1$ (3%)<br>...	$t_{2\mathrm{g}}^1t_{2\mathrm{g}}^2t_{2\mathrm{g}}^2t_{2\mathrm{g}}^2$ (89%)<br>$t_{2\mathrm{g}}^1t_{2\mathrm{g}}^2t_{2\mathrm{g}}^1t_{2\mathrm{g}}^3$ (9%)<br>...
AlV_4S_8	$a_1^2e^4t_2^1t_1^0t_2^0$ (19%)<br>$a_1^1e^3t_2^3t_1^0t_2^0$ (6%)<br>$a_1^1e^4t_2^2t_1^0t_2^0$ (3%)<br>...	$t_{2\mathrm{g}}^1t_{2\mathrm{g}}^2t_{2\mathrm{g}}^2t_{2\mathrm{g}}^2$ (89%)<br>$t_{2\mathrm{g}}^1t_{2\mathrm{g}}^2t_{2\mathrm{g}}^1t_{2\mathrm{g}}^3$ (9%)<br>...	$GaNb_4S_8$	$a_1^2e^4t_2^1t_1^0t_2^0$ (66%)<br>$a_1^2e^2t_2^3t_1^0t_2^0$ (5%)<br>$a_1^2e^2t_2^2t_1^1t_2^0$ (3%)<br>...	$t_{2\mathrm{g}}^1t_{2\mathrm{g}}^2t_{2\mathrm{g}}^2t_{2\mathrm{g}}^2$ (54%)<br>$t_{2\mathrm{g}}^1t_{2\mathrm{g}}^2t_{2\mathrm{g}}^1t_{2\mathrm{g}}^3$ (36%)<br>$t_{2\mathrm{g}}^0t_{2\mathrm{g}}^2t_{2\mathrm{g}}^2t_{2\mathrm{g}}^3$ (5%)<br>...
$GaNb_4Se_8$	$a_1^2e^4t_2^1t_1^0t_2^0$ (64%)<br>$a_1^2e^2t_2^3t_1^0t_2^0$ (6%)<br>$a_1^2e^2t_2^2t_1^1t_2^0$ (3%)<br>...	$t_{2\mathrm{g}}^1t_{2\mathrm{g}}^2t_{2\mathrm{g}}^2t_{2\mathrm{g}}^2$ (56%)<br>$t_{2\mathrm{g}}^1t_{2\mathrm{g}}^2t_{2\mathrm{g}}^1t_{2\mathrm{g}}^3$ (36%)<br>$t_{2\mathrm{g}}^1t_{2\mathrm{g}}^3t_{2\mathrm{g}}^0t_{2\mathrm{g}}^3$ (7%)<br>...	$GaTa_4Se_8$	$a_1^2e^4t_2^1t_1^0t_2^0$ (71%)<br>$a_1^2e^2t_2^3t_1^0t_2^0$ (6%)<br>$a_1^2e^2t_2^2t_1^1t_2^0$ (3%)<br>...	$t_{2\mathrm{g}}^1t_{2\mathrm{g}}^2t_{2\mathrm{g}}^2t_{2\mathrm{g}}^2$ (48%)<br>$t_{2\mathrm{g}}^1t_{2\mathrm{g}}^2t_{2\mathrm{g}}^1t_{2\mathrm{g}}^3$ (39%)<br>$t_{2\mathrm{g}}^1t_{2\mathrm{g}}^1t_{2\mathrm{g}}^1t_{2\mathrm{g}}^4$ (9%)<br>...

IV. SUPPLEMENTARY TABLE: CALCULATED DYNAMICAL I_{D} AND NONDYNAMICAL I_{ND} INDICES

In the following Supplementary Table 7, the natural orbital occupation numbers (NOOC) for the (7e,12o)-CASSCF are given. From these, using the formula proposed by Ramos-Cordoba *et al.* [27] the dynamical I_{D} and nondynamical I_{ND} indices were calculated from the NOOC of each molecular orbital i:

$$n_i = \mathrm{NOOC}_i/2.0 \tag{1}$$

$$I_{\mathrm{D}} = 2 \cdot \left[\left(\frac{1}{4} \sum_i \sqrt{n_i \cdot (1 - n_i)} \right) - \left(\frac{1}{2} \sum_i n_i \cdot (1 - n_i) \right) \right] \tag{2}$$

$$I_{\mathrm{ND}} = 2 \cdot \left[\left(\frac{1}{2} \sum_i n_i \cdot (1 - n_i) \right) \right] \tag{3}$$

From the NOOCs as well as the I_{ND} values it becomes clear that the seven-electron active space inherits stronger nondynamical correlation effects in the $4d$ vanadates as compared to the $4d$ and $5d$ analogues. Still, even in the latter two classes of compounds, the nondynamical correlation is substantial and adds up to about 60% of the summed correlation index.

Supplementary Table 7. Natural orbital occupation numbers (NOOC) and dynamical I_{D} and nondynamical I_{ND} indices of the $^2T_2$ ground state term in the investigated group-5 lacunar spinels.

Compound	NOOC (a_1, e, t_2, t_1, t_2)	I_{D}	I_{ND}	Compound	NOOC (a_1, e, t_2, t_1, t_2)	I_{D}	I_{ND}
GaV_4S_8	1.30, 1.20, 0.66, 0.22, 0.22	0.41	1.96	GaV_4Se_8	1.27, 1.15, 0.69, 0.22, 0.23	0.40	2.00
AlV_4S_8	1.26, 1.15, 0.70, 0.22, 0.22	0.40	2.00	$GaNb_4S_8$	1.81, 1.73, 0.43, 0.09, 0.07	0.63	1.05
$GaNb_4Se_8$	1.81, 1.71, 0.43, 0.09, 0.07	0.63	1.07	$GaTa_4Se_8$	1.86, 1.78, 0.42, 0.06, 0.05	0.62	0.92

V. SUPPLEMENTARY FIGURE: CALCULATED SUSCEPTIBILITY CURVES

Experimental susceptibility measurements show deviations from Curie-Weiss law for GaV_4S_8 [3, 28], which we attribute to low-energy gap between ground $^2T_2$ and ^4T excited states (41 meV). To further illustrate this point, the $1/\chi$ susceptibility curves were calculated for the three lacunar spinels GaV_4S_8, $GaNb_4Se_8$ and $GaTa_4Se_8$ (see Supplementary Fig. 2). For all three compounds, this deviation from Curie-Weiss law is observed here. As expected from the increasing effect of SOC in the order $3d < 4d < 5d$, the maximum inverse susceptibility increases, too. However, quantitative agreement with experiment is not reached, e.g. for GaV_4S_8 the curve is expected to saturate at a value of ≈900 mol/emu [28], while we find 350 mol/emu. A possible source of error are missing vibronic and intersite magnetic coupling effects in our simulations. For $GaNb_4Se_8$ and $GaTa_4Se_8$, the contribution of the former was studied based on calculated g factors and associated effective magnetic moments along the Jahn-Teller distortion pathway [19]. A significant effect of this distortion was found, which leads to increasing g factors and effective magnetic moments. Eventually, g factors in closer agreement to experiment are found for these distorted structures.

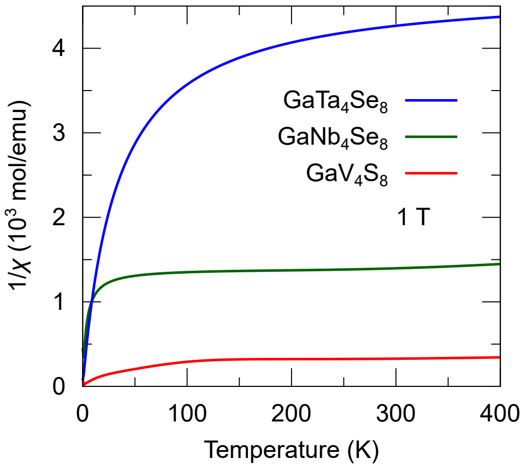

Supplementary Figure 2. Calculated $1/\chi$ susceptibility curves for GaV_4S_8 (red), $GaNb_4Se_8$ (green) and $GaTa_4Se_8$ (blue). The curves are simulated using the SINGLE_ANISO suite [29] on top of the spin-orbit (SO) eigenstates shown in Fig. 2 of the main manuscript (i.e. for GaV_4S_8 NEVPT2(7e,12o)+SO and for $GaNb_4Se_8$/$GaTa_4Se_8$ CASSCF(7e,12o)+SO values were taken as a reference).

SUPPLEMENTARY REFERENCES

[1] M. Klintenberg, S. Derenzo, and M. Weber, Accurate crystal fields for embedded cluster calculations, Comp. Phys. Commun. **131**, 120 (2000).

[2] S. E. Derenzo, M. K. Klintenberg, and M. J. Weber, Determining point charge arrays that produce accurate ionic crystal fields for atomic cluster calculations, J. Chem. Phys. **112**, 2074 (2000).

[3] A. Štefančič, S. J. Holt, M. R. Lees, C. Ritter, M. J. Gutmann, T. Lancaster, and G. Balakrishnan, Establishing magneto-structural relationships in the solid solutions of the skyrmion hosting family of materials: $GaV_4S_{8-y}Se_y$, Sci. Rep. **10**, 9813 (2020).

[4] D. Bichler, Magnetismus und strukturelle Phasenumwandlungen von Verbindungen mit tetraedrischen Metallclustern, PhD thesis, Ludwigs-Maximilians-Universität München, Germany (2010).

[5] S. Jakob, H. Müller, D. Johrendt, S. Altmannshofer, W. Scherer, S. Rayaprol, and R. Pöttgen, Structural and magnetic transitions in the Mott insulator $GaNb_4S_8$, J. Mater. Chem. **17**, 3833 (2007).

[6] R. Pocha, D. Johrendt, B. Ni, and M. M. Abd-Elmeguid, Crystal Structures, Electronic Properties, and Pressure-Induced Superconductivity of the Tetrahedral Cluster Compounds $GaNb_4S_8$, $GaNb_4Se_8$, and $GaTa_4Se_8$, J. Amer. Chem. Soc. **127**, 8732 (2005).

[7] S. R. Cox and D. E. Williams, Representation of the molecular electrostatic potential by a net atomic charge model, J. Comput. Chem. **2**, 304 (1981).

[8] C. M. Breneman and K. B. Wiberg, Determining atom-centered monopoles from molecular electrostatic potentials. The need for high sampling density in formamide conformational analysis, J. Comput. Chem. **11**, 361 (1990).

[9] K. B. Wiberg and P. R. Rablen, Comparison of atomic charges derived via different procedures, J. Comput. Chem. **14**, 1504 (1993).

[10] A. Dittmer, G. L. Stoychev, D. Maganas, A. A. Auer, and F. Neese, Computation of NMR Shielding Constants for Solids Using an Embedded Cluster Approach with DFT, Double-Hybrid DFT, and MP2, J. Chem. Theor. Comput. **16**, 6950 (2020).

[11] M. Dolg, U. Wedig, H. Stoll, and H. Preuss, Energy-adjusted ab initio pseudopotentials for the first row transition elements, J. Chem. Phys. **86**, 866 (1987).

[12] D. Andrae, U. Häußermann, M. Dolg, H. Stoll, and H. Preuß, Energy-adjusted ab initio pseudopotentials for the second and third row transition elements, Theoret. Chim. Acta **77**, 123 (1990).

[13] A. Bergner, M. Dolg, W. Küchle, H. Stoll, and H. Preuß, Ab initio energy-adjusted pseudopotentials for elements of groups 13–17, Mol. Phys. **80**, 1431 (1993).

[14] T. Leininger, A. Berning, A. Nicklass, H. Stoll, H.-J. Werner, and H.-J. Flad, Spin-orbit interaction in heavy group 13 atoms and TlAr, Chem. Phys. **217**, 19 (1997).

[15] B. O. Roos, The complete active space self-consistent field method and its applications in electronic structure calculations, in *Adv. Chem. Phys.* (John Wiley & Sons, Ltd, 1987) pp. 399–445.

[16] C. Angeli, R. Cimiraglia, S. Evangelisti, T. Leininger, and J.-P. Malrieu, Introduction of n-electron valence states for multireference perturbation theory, J. Chem. Phys. **114**, 10252 (2001).

[17] Y. Wang, D. Puggioni, and J. M. Rondinelli, Assessing exchange-correlation functional performance in the chalcogenide lacunar spinels $\mathrm{Ga}M_4Q_8$ ($M = \mathrm{Mo, V, Nb, Ta}$; $Q = \mathrm{S, Se}$), Phys. Rev. B **100**, 115149 (2019).

[18] H. Lee, M. Y. Jeong, J.-H. Sim, H. Yoon, S. Ryee, and M. J. Han, Charge density functional plus U calculation of lacunar spinel GaM_4Se_8 (M = Nb, Mo, Ta, and W), Europhys. Lett. **125**, 47005 (2019).

[19] T. Petersen, L. Prodan, V. Tsurkan, H.-A. Krug von Nidda, I. Kézsmárki, U. K. Rößler, and L. Hozoi, How Correlations and Spin–Orbit Coupling Work within Extended Orbitals of Transition-Metal Tetrahedra of 4d/5d Lacunar Spinels, J. Phys. Chem. Lett. **13**, 1681 (2022).

[20] M. Douglas and N. M. Kroll, Quantum electrodynamical corrections to the fine structure of helium, Ann. Phys. **82**, 89 (1974).

[21] B. A. Hess, Relativistic electronic-structure calculations employing a two-component no-pair formalism with external-field projection operators, Phys. Rev. A **33**, 3742 (1986).

[22] N. B. Balabanov and K. A. Peterson, Systematically convergent basis sets for transition metals. I. All-electron correlation consistent basis sets for the 3d elements Sc-Zn, J. Chem. Phys. **123**, 064107 (2005).

[23] W. A. de Jong, R. J. Harrison, and D. A. Dixon, Parallel Douglas-Kroll energy and gradients in NWChem: Estimating scalar relativistic effects using Douglas-Kroll contracted basis sets, J. Chem. Phys. **114**, 48 (2001).

[24] J. D. Rolfes, F. Neese, and D. A. Pantazis, All-electron scalar relativistic basis sets for the elements Rb–Xe, J. Comput. Chem. **41**, 1842 (2020).

[25] F. Weigend and R. Ahlrichs, Balanced basis sets of split valence, triple zeta valence and quadruple zeta valence quality for H to Rn: Design and assessment of accuracy, Phys. Chem. Chem. Phys. **7**, 3297 (2005).

[26] D. A. Pantazis, X.-Y. Chen, C. R. Landis, and F. Neese, All-electron scalar relativistic basis sets for third-row transition metal atoms, J. Chem. Theor. Comput. **4**, 908 (2008).

[27] E. Ramos-Cordoba, P. Salvador, and E. Matito, Separation of dynamic and nondynamic correlation, Phys. Chem. Chem. Phys. **18**, 24015 (2016).
[28] S. Widmann, E. Ruff, A. Günther, H.-A. K. von Nidda, P. Lunkenheimer, V. Tsurkan, S. Bordács, I. Kézsmárki, and A. Loidl, On the multiferroic skyrmion-host GaV_4S_8, Philos. Mag. **97**, 3428 (2017).
[29] L. Chibotaru and L. Ungur, Ab initio calculation of anisotropic magnetic properties of complexes. I. Unique definition of pseudospin Hamiltonians and their derivation, J. Chem. Phys. **137**, 064112 (2012).
