# Supplementary material for: Resonating holes vs molecular spin-orbit coupled states in group-5 lacunar spinels
Source: Nat Commun. 2023 Aug 26;14:5218. doi: 10.1038/s41467-023-40811-y (PMC10460446; doi:10.1038/s41467-023-40811-y)
Supplement: Supplementary file 3 — Description of Additional Supplementary Files [file 41467_2023_40811_MOESM3_ESM.pdf]

Description of Additional Supplementary Files

Supplementary Dataset 1: Coordinates of quantum cluster and point charge field.
